# Modified Blalock-Thomas-Taussig Shunt Using Femoral Artery Homograft

**DOI:** 10.1016/j.atssr.2022.11.004

**Published:** 2022-11-09

**Authors:** Sandy Zhang, Suneet Bhansali, Jaclyn McKinstry, Prema Ramaswamy, Kristen Thomas, Michael Martinez, Ralph S. Mosca, T.K. Susheel Kumar

**Affiliations:** 1 Department of Pediatrics, NYU Grossman School of Medicine, NYU Langone Health, New York, New York; 2 Division of Pediatric Cardiology, NYU Grossman School of Medicine, NYU Langone Health, New York, New York; 3 Division of Pediatric Cardiology, Maimonides Medical Center, Brooklyn, New York; 4 Department of Pathology, NYU Grossman School of Medicine, NYU Langone Health, New York, New York; 5 Department of Congenital Cardiothoracic Surgery, NYU Grossman School of Medicine, NYU Langone Health, New York, New York

## Abstract

Modified Blalock-Thomas-Taussig shunts are typically performed with biosynthetic polytetrafluoroethylene grafts. However, biologic conduits are being increasingly investigated. We herein report a case in which a femoral artery homograft was effectively used as material for a Blalock-Thomas-Taussig shunt.

Thrombosis is a known complication of the modified Blalock-Thomas-Taussig shunt (BTTS) with use of the standard polytetrafluoroethylene (PTFE) graft.^1^ Patients with cyanotic congenital heart defects and concurrent sickle cell disease are at an increased risk of thrombosis.^2^ We herein report a case of BTTS placement using a femoral artery homograft in an infant with tetralogy of Fallot and critical pulmonary stenosis who also had sickle cell disease.

A 3-month-old boy with sickle cell disease and tetralogy of Fallot with severe multilevel pulmonary stenosis and confluent branch pulmonary arteries was transferred to our institution for management of hypoxia. Hemoglobin electrophoresis revealed a pattern of 21% hemoglobin S and 78% hemoglobin F. His oxygen saturations were 50% to 60% on room air. Transthoracic echocardiography demonstrated a peak instantaneous gradient of 75 mm Hg across the right ventricular outflow tract, trivial patent ductus arteriosus, and possible dual left anterior descending coronary arteries.

Intraoperatively, the pulmonary valve and main pulmonary artery segment were noted to be hypoplastic. Clockwise rotation of the right coronary artery and a prominent conal branch crossing the right ventricular outflow tract were also observed. Hence, the decision was made to pursue palliation with modified BTTS placement. A 4.0-mm cadaveric femoral artery homograft was thawed and introduced into the field. An appropriate segment was tailored and beveled. The beveled end was anastomosed to the inferior aspect of the innominate artery by a continuous 7-0 Prolene suture. The other end was then tailored and anastomosed to the anterosuperior aspect of the right branch pulmonary artery again by a continuous 7-0 Prolene suture. On release of the clamp, an immediate drop in perfusion pressure was noted, indicating good flow throughout the shunt. The patient was successfully weaned off cardiopulmonary bypass with stable hemodynamics and oxygen saturations of 80% to 90%. There was minimal bleeding at the suture lines. Transesophageal echocardiography demonstrated a widely patent BTTS with good bilateral pulmonary venous return and normal biventricular systolic function.

The patient did well postoperatively, and he was discharged home without any significant clinical events. Echocardiography before discharge confirmed a patent BTTS. At subsequent outpatient visits, he demonstrated adequate saturations on room air and appropriate weight gain.

At 1 year of age, he underwent complete repair of his intracardiac defect after exchange transfusion on cardiopulmonary bypass. During the operation, the femoral artery homograft was noted to be widely patent with minimal adhesions and easy to dissect (Figure 1). His subsequent postoperative course was uncomplicated. Histopathologic examination of the femoral artery homograft was notable for fibrous expansion of the intima with no evidence of thrombosis (Figure 2).

**Figure 1:**
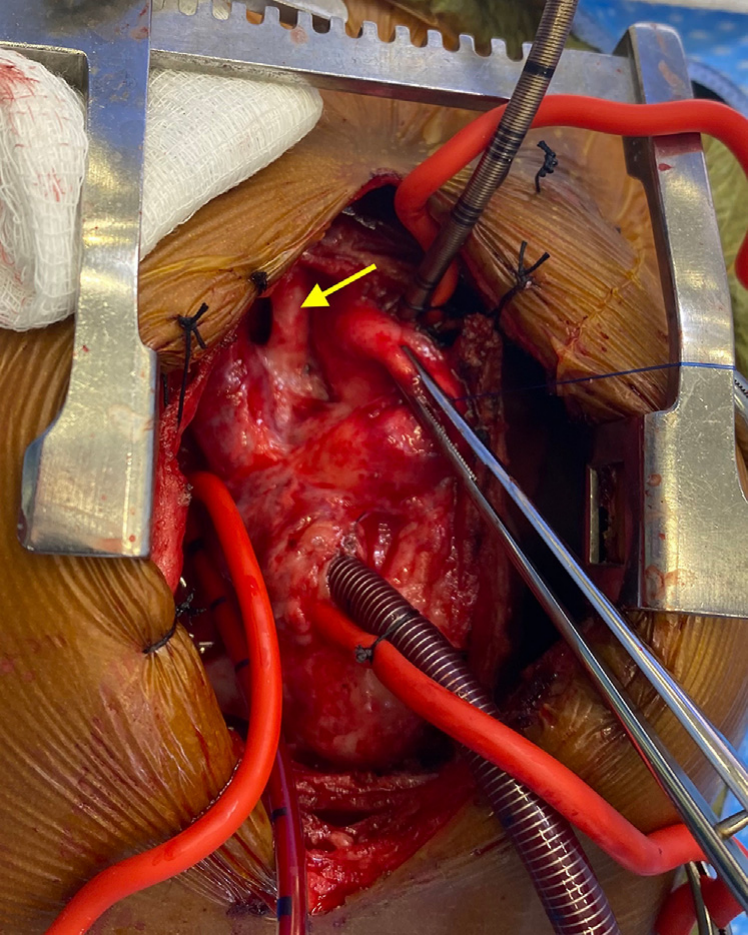
Blalock-Thomas-Taussig shunt using a femoral artery homograft (arrow) as visualized during complete repair of tetralogy of Fallot.

**Figure 2:**
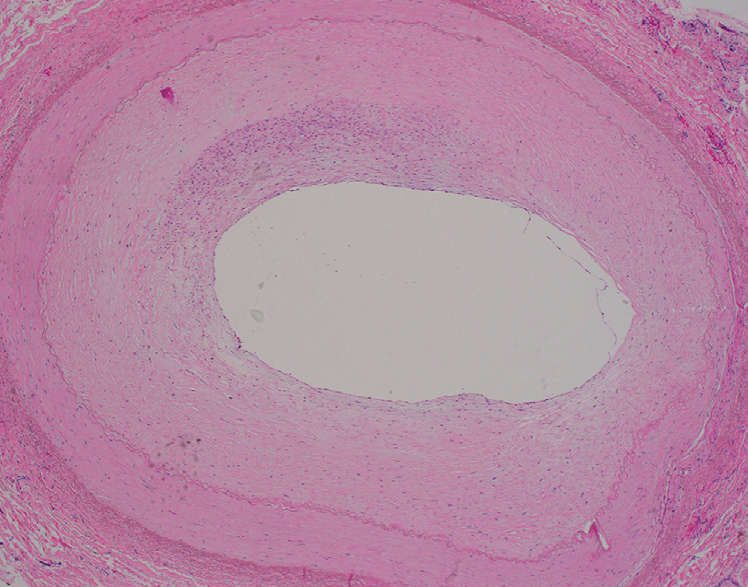
Histopathology of the femoral artery homograft specimen (hematoxylin and eosin staining, magnification ×4).

## Comment

The classic BTTS was first described by Helen Taussig, Alfred Blalock, and Vivian Thomas in the 1940s. Since then, many variations of the classic shunt have been described, of which the modified BTTS with use of biosynthetic PTFE is the most common.^3^ The modified BTTS introduces a conduit between the subclavian artery and the pulmonary artery, rather than a direct anastomosis. Shunt thrombosis, however, remains a serious complication of the modified BTTS, ranging from 8% to 22%.^1^ Risk factors for shunt thrombosis include younger age and smaller shunt size.^3^ The diagnosis of sickle cell disease in our patient presented an additional risk factor for thrombosis and subsequent shunt failure.^2^ However, we were successfully able to palliate our patient for 9 months using a femoral artery homograft BTTS without complications.

The conduit choice for a modified BTTS is typically PTFE; however, biologic conduits, such as saphenous vein homografts, are being increasingly investigated.^1^ Biologic conduits have many theoretical advantages, including endothelial lining that may protect against thrombosis and infection, intrinsic hemostatic properties, and better distensibility, allowing longevity.^4^ In a retrospective review of 136 patients, Kaur and associates^1^ found that saphenous vein grafts result in significantly less thrombosis compared with PTFE grafts. At our institute, we commonly use saphenous vein grafts for construction of BTTS in neonates; however, an appropriately sized graft was not immediately available, and a femoral artery graft was used instead. A thorough review of literature did not reveal any prior reports of use of femoral artery homograft as a systemic-pulmonary artery shunt. These grafts have been used in cardiac operations for coronary artery bypass grafting as well as for repair of coronary artery stenosis.^5^ Further studies are needed to evaluate the role of arterial homografts in modified BTTS, but our case presents one instance in which they may be a safe alternative.
